# Supplementary material for: Hfq CLASH uncovers sRNA-target interaction networks linked to nutrient availability adaptation
Source: eLife. 2020 May 1;9:e54655. doi: 10.7554/eLife.54655 (PMC7213987; doi:10.7554/eLife.54655)
Supplement: Supplementary file 11. [file elife-54655-supp11.docx]

**Supplementary File 11**

| **Key Resources Table** | | | | |
| --- | --- | --- | --- | --- |
| **Reagent type (species) or resource** | **Designation** | **Source or reference** | **Identifiers** | **Additional information** |
| strain, strain background (*Escherichia coli*) | MG1655 *hfq::htf* | (Tree et al., 2014) |  | Hfq tagged strain for CLASH |
| strain, strain background (*Escherichia coli*) | MG1655 | (Blattner et al., 1997) |  | Parental control strain |
| strain, strain background (*Escherichia coli*) | TOP10 F’ | Invitrogen |  | For co-expression of sRNA plasmids |
| recombinant DNA reagent | pZA21MCS (plasmid) | Expressys |  | Backbone for pZA-sRNA plasmids |
| recombinant DNA reagent | pZA21 (plasmid) | (Tree et al., 2014) |  | pZA21MCS with MCS removed from +1 to terminator |
| recombinant DNA reagent | pZA21::CyaR (plasmid) | This paper |  | aTC-inducible expression of CyaR |
| recombinant DNA reagent | pZA21::CyaR 38-39  (plasmid) | This paper |  | CyaR C38 and C39 residues mutated to GG |
| recombinant DNA reagent | pZA21::ArcZ (plasmid) | This paper |  | aTC-inducible expression of ArcZ |
| recombinant DNA reagent | pZE12luc (plasmid) | Expressys |  | Backbone for pZE-sRNA plasmids |
| recombinant DNA reagent | pJV300 (plasmid) | (Sittka et al., 2007) |  | sRNA control plasmid for pZE12 expressed sRNAs |
| recombinant DNA reagent | pZE12::ArcZ (plasmid) | This paper |  | IPTG-inducible expression of ArcZ |
| recombinant DNA reagent | pZE12::ArcZ 70-71 (plasmid) | This paper |  | ArcZ G70 and G71 residues mutated to CC |
| recombinant DNA reagent | pZE12::CyaR (plasmid) | This paper |  | IPTG-inducible expression of CyaR |
| antibody | anti-TAP (Rabbit polyclonal) | Thermo Fisher | Cat. # CAB1001, RRID:AB_10709700 | 1:5000 in blocking solution, 4°C, overnight |
| antibody | anti-GroEL (Rabbit polyclonal) | Abcam | Cat. # ab82592, RRID:AB_1658428 | 1:150000 in blocking solution, room temperature, 2h |
| antibody | Anti-rabbit IgG H&L (IRDye 800CW) (Goat polyclonal) | Abcam | Cat# ab216773 | 1:10000 in blocking solution, room temperature, 1h |
| sequence-based reagent | L5Aa | IDT | CLASH L5 5’adapter | 5'-invddT-ACACrGrArCrGrCrUrCrUrUrCrCrGrArUrCrUrNrNrNrUrArArGrCrN-OH-3' |
| sequence-based reagent | L5Ab | IDT | CLASH L5 5’adapter | 5'-invddT-ACACrGrArCrGrCrUrCrUrUrCrCrGrArUrCrUrNrNrNrArUrUrArGrCrN-OH-3' |
| sequence-based reagent | L5Ac | IDT | CLASH L5 5’adapter | 5'-invddT-ACACrGrArCrGrCrUrCrUrUrCrCrGrArUrCrUrNrNrNrGrCrGrCrArGrCrN-OH-3' |
| sequence-based reagent | L5Ad | IDT | CLASH L5 5’adapter | 5'-invddT-ACACrGrArCrGrCrUrCrUrUrCrCrGrArUrCrUrNrNrNrCrGrCrUrUrArGrCrN-OH-3' |
| sequence-based reagent | L5Ba | IDT | CLASH L5 5’adapter | 5'-invddT-ACACrGrArCrGrCrUrCrUrUrCrCrGrArUrCrUrNrNrNrArGrArGrCrN-OH-3' |
| sequence-based reagent | L5Bb | IDT | CLASH L5 5’adapter | 5'-invddT-ACACrGrArCrGrCrUrCrUrUrCrCrGrArUrCrUrNrNrNrGrUrGrArGrCrN-OH-3' |
| sequence-based reagent | L5Bc | IDT | CLASH L5 5’adapter | 5'-invddT-ACACrGrArCrGrCrUrCrUrUrCrCrGrArUrCrUrNrNrNrCrArCrUrArGrCrN-OH-3' |
| sequence-based reagent | L5Bd | IDT | CLASH L5 5’adapter | 5'-invddT-ACACrGrArCrGrCrUrCrUrUrCrCrGrArUrCrUrNrNrNrUrCrUrCrUrArGrCrN-OH-3' L5b |
| sequence-based reagent | L5Ca | IDT | CLASH L5 5’adapter | 5'-invddT-ACACrGrArCrGrCrUrCrUrUrCrCrGrArUrCrUrNrNrNrCrUrArGrCrN-OH-3' |
| sequence-based reagent | L5Cb | IDT | CLASH L5 5’adapter | 5'-invddT-ACACrGrArCrGrCrUrCrUrUrCrCrGrArUrCrUrNrNrNrUrGrGrArGrCrN-OH-3' |
| sequence-based reagent | L5Cc | IDT | CLASH L5 5’adapter | 5'-invddT-ACACrGrArCrGrCrUrCrUrUrCrCrGrArUrCrUrNrNrNrArCrUrCrArGrCrN-OH-3' |
| sequence-based reagent | L5Cd | IDT | CLASH L5 5’adapter | 5'-invddT-ACACrGrArCrGrCrUrCrUrUrCrCrGrArUrCrUrNrNrNrGrArCrUrUrArGrCrN-OH-3' |
| sequence-based reagent | L5Da | IDT | CLASH L5 5’adapter | 5'-invddT-ACACrGrArCrGrCrUrCrUrUrCrCrGrArUrCrUrNrNrNrCrGrUrGrArUrN-OH-3' |
| sequence-based reagent | L5Db | IDT | CLASH L5 5’adapter | 5'-invddT-ACACrGrArCrGrCrUrCrUrUrCrCrGrArUrCrUrNrNrNrGrCrArCrUrArN-OH-3' |
| sequence-based reagent | L5Dc | IDT | CLASH L5 5’adapter | 5'-invddT-ACACrGrArCrGrCrUrCrUrUrCrCrGrArUrCrUrNrNrNrUrArGrUrGrCrN-OH-3' |
| sequence-based reagent | L5Dd | IDT | CLASH L5 5’adapter | 5'-invddT-ACACrGrArCrGrCrUrCrUrUrCrCrGrArUrCrUrNrNrNrArUrCrArCrGrN-OH-3' |
| sequence-based reagent | L5Ea | IDT | CLASH L5 5’adapter | 5'-invddT-ACACrGrArCrGrCrUrCrUrUrCrCrGrArUrCrUrNrNrNrCrArCrUrGrUrN-OH-3' |
| sequence-based reagent | L5Eb | IDT | CLASH L5 5’adapter | 5'-invddT-ACACrGrArCrGrCrUrCrUrUrCrCrGrArUrCrUrNrNrNrGrUrGrArCrArN-OH-3' |
| sequence-based reagent | L5Ec | IDT | CLASH L5 5’adapter | 5'-invddT-ACACrGrArCrGrCrUrCrUrUrCrCrGrArUrCrUrNrNrNrUrGrUrCrArCrN-OH-3' |
| sequence-based reagent | L5Ed | IDT | CLASH L5 5’adapter | 5'-invddT-ACACrGrArCrGrCrUrCrUrUrCrCrGrArUrCrUrNrNrNrArCrArGrUrGrN-OH-3' |
| sequence-based reagent | App_PE | IDT | CLASH 3’adapter | 5’App-NAGATCGGAAGAGCACACGTCTG-ddC-3’ |
| sequence-based reagent | PE_reverse | IDT | RT primer | 5’-CAGACGTGTGCTCTTCCGATCT-3’ |
| sequence-based reagent | PE_Solexa_Hexamer | IDT | RT primer for making RNA-Seq libraries | 5’-CGTGTGCTCTTCCGATCTNNNNNN-3’ |
| sequence-based reagent | P5_phospho_adapter | IDT | RNA-Seq adapter for ligating to 3’ ends of cDNAs | 5’P-AGATCGGAAGAGCGTCGTGTAGGG-3SpC3 |
| sequence-based reagent | P5 | IDT | PCR oligonucleotide (Forward) | 5’-AATGATACGGCGACCACCGAGATCTACACTCTTTCCCTACACGACGCTCTTCCGATCT-3’ |
| sequence-based reagent | BC1 | IDT | P3 PCR oligonucleotide (Reverse) | 5’-CAAGCAGAAGACGGCATACGAGATCGTGATGTGACTGGAGTTCAGACGTGTGCTCTTCCGATCT-3’ |
| sequence-based reagent | BC2 | IDT | P3 PCR oligonucleotide (Reverse) | 5’-CAAGCAGAAGACGGCATACGAGATACATCGGTGACTGGAGTTCAGACGTGTGCTCTTCCGATCT-3’ |
| sequence-based reagent | BC3 | IDT | P3 PCR oligonucleotide (Reverse) | 5’-CAAGCAGAAGACGGCATACGAGATGCCTAAGTGACTGGAGTTCAGACGTGTGCTCTTCCGATCT-3’ |
| sequence-based reagent | BC4 | IDT | P3 PCR oligonucleotide (Reverse) | 5’-CAAGCAGAAGACGGCATACGAGATTGGTCAGTGACTGGAGTTCAGACGTGTGCTCTTCCGATCT-3’ |
| sequence-based reagent | BC5 | IDT | P3 PCR oligonucleotide (Reverse) | 5’-CAAGCAGAAGACGGCATACGAGATCACTGTGTGACTGGAGTTCAGACGTGTGCTCTTCCGATCT-3’ |
| sequence-based reagent | BC6 | IDT | P3 PCR oligonucleotide (Reverse) | 5’-CAAGCAGAAGACGGCATACGAGATATTGGCGTGACTGGAGTTCAGACGTGTGCTCTTCCGATCT-3’ |
| other | Vari-X-link | van Nues et al., 2017 | https://www.vari-x-link.com | UV cross-linking. Essential for fast cross-linking of cultures. |
| other | Nitrocellulose filters (0.45 µM) | Sigma | Cat. #: HAWP14250 | Cell harvesting |
| chemical compound, drug | Protease inhibitors | Roche | Cat. #: A32965 |  |
| other | Zirconia beads (0.1 mm) | Thistle Scientific | Cat. #: 11079101z | Cell lysis |
| chemical compound, drug | RQ1 DNase I (10U/ml) | Promega | Cat. #: M6101 | DNase treatment of lysate |
| other | M2 anti-FLAG beads | Sigma | Cat. #: M8823-5ML | For immunoprecipitation |
| chemical compound, drug | GST-TEV protease | other |  | Home-made. Quantity depends on activity of batch |
| chemical compound, drug | RnaceIt (RNase A and T1 mix) | Agilent | Cat. #: 400720 | Trimming of cross-linked RNAs |
| chemical compound, drug | GuHCl | Sigma | Cat. #: G3272-100G | 6M |
| chemical compound, drug | Imidazole | Sigma | Cat. #: I202-25G |  |
| other | Nichel-NTA agarose beads | Qiagen | Cat. #: 30210 |  |
| other | Pierce SnapCap spin columns | Thermo Fisher | Cat. #: 69725 |  |
| chemical compound, drug | TSAP (Thermosensitive alkaline phosphatase) | Promega | Cat. #: M9910 | For dephosphorylating cross-linked RNA |
| chemical compound, drug | RNasin | Promega | Cat. #: N2115 | RNase inhibitor |
| chemical compound, drug | Truncated T4 RNA ligase 2 K227Q | NEB | Cat. #: M0351L | For ligating 5’ adenylated adapter to 3’ end of cross-linked RNA |
| chemical compound, drug | T4 PNK | NEB | Cat. #: M0201L | For phosphorylating 5’ ends of cross-linked RNA |
| chemical compound, drug | ^32^P-γATP | Perkin Elmer | Cat. #: NEG502Z-500 | For radioactively labeling the 5’ end of the cross-linked RNA using T4 PNK |
| chemical compound, drug | ATP | Roche | Cat. #: 11140965001 | For phosphorylating 5’ ends of cross-linked RNAs |
| chemical compound, drug | T4 RNA Ligase 1 | NEB | Cat. #: M0204L | For ligating 5’ CRAC adapters to cross-linked RNAs |
| chemical compound, drug | TCA | SIGMA | Cat. #: T0699-100ML | For precipitating protein-RNA complex |
| other | NuPAGE loading buffer | Thermo Scientific | Cat. #: NP0007 |  |
| other | NuPAGE gel | Thermo Scientifc | Cat. #: NP0323PK2 |  |
| chemical compound, drug | Proteinase K | Roche | Cat. #: 3115801001 |  |
| chemical compound, drug | Superscript IV | Invitrogen | Cat. #: 18090010 | For reverse transcribing cross-linked RNAs |
| commercial assay or kit | DNA Clean and Concentrator 5 kit | Zymo Research | Cat. #: D4003T |  |
| chemical compound, drug | Pfu Polymerase | Promega | Cat. #: M7745 |  |
| chemical compound, drug | Exonuclease I | NEB | Cat. #: M0293L | For removing free DNA oligonucleotides from the PCR reactions |
| chemical compound, drug | MetaPhor agarose | Lonza | Cat. #: LZ50181 |  |
| commercial assay or kit | MinElute kit | Qiagen | Cat. #: 28004 |  |
| other | 2100 Bioanalyzer | Agilent | Cat. #: G2939BA |  |
| other | Qubit 4 | Thermo Scientifc | Cat. #: Q33226 |  |
| chemical compound, drug | IPTG | Sigma | Cat. #: I6758-1G | 1 mM |
| chemical compound, drug | aTC (anhydrotetracycline hydrochloride) | Sigma | Cat. #: 1035708-25MG | 200 nM |
| chemical compound, drug | Turbo DNase | Thermo Scientfic | Cat. #: AM2238 | For removing contaminating DNA in RNA samples |
| chemical compound, drug | SuperaseIn RNase inhibitor | Thermo Scientific | Cat. #: AM2694 |  |
| commercial assay or kit | RNAClean XP beads | Beckman Coulter | Cat. #: A63987 |  |
| commercial assay or kit | Luna Universal One-Step RT-qPCR Kit | NEB | Cat. #: E3005E | Quantitative PCR kit used in this study |
| other | HyBond N+ nylon membrane | GEHealthcare | Cat. #: RPN1210B |  |
| other | UltraHyb Oligo Hyb | Thermo Scientific | Cat. #: AM8663 | For Northern blot analysis |
| sequence-based reagent | RT-qPCR oligos | IDT | RT and qPCR primers | See Supplementary File 10 (Oligos) |
| sequence-based reagent | Northern Blot probes | IDT | DNA oligos | See Supplementary File 10 (Oligos) |
| sequence-based reagent | Ultramers | IDT | PCR primers for cloning | See Supplementary File 10 (Oligos) |
| software, algorithm | CRAC_pipeline, pyCRAC and Genome Browser packages | (Webb et al., 2014) | <https://bitbucket.org/sgrann>;  <https://git.ecdf.ed.ac.uk/sgrannem> | Software packages and data analysis pipelines used in this study. |
| software, algorithm | FLASH | (Magoč and Salzberg, 2011) | <https://github.com/dstreett/FLASH2> |  |
| software, algorithm | hyb | (Travis et al., 2013) | <https://github.com/gkudla/hyb> |  |
| software, algorithm | hyb_stats | (Waters et al., 2017) | <https://bitbucket.org/jaitree/hyb_stats/> |  |
| software, algorithm | BEDtools | (Quinlan and Hall, 2010) | <https://github.com/arq5x/bedtools2/releases> |  |
| software, algorithm | Image Studio | LI-COR Biosciences | https://www.licor.com/bio/image-studio-lite |  |
